# Primary splenic epithelioid hemangioendothelioma with diffuse metastases revealed by FDG PET/CT imaging: A case report

**DOI:** 10.1097/MD.0000000000025065

**Published:** 2021-04-02

**Authors:** Xian Li, Xiaowei Ma, Jingwen Hao, Chuning Dong, Yunhua Wang

**Affiliations:** Department of Nuclear Medicine/PET Center, The Second Xiangya Hospital, Central South University, Changsha, Hunan, China.

**Keywords:** case report, epithelioid hemangioendothelioma, fluorodeoxyglucose, metastasis, PET/CT, positron emission tomography/computed tomography, spleen

## Abstract

**Rationale:**

Epithelioid hemangioendothelioma (EHE) is a rare low-to-intermediate grade malignant vascular neoplasm. We report a primary splenic EHE with diffused metastasis who underwent ^18^F-fluorodeoxyglucose positron emission tomography/computed tomography (FDG PET/CT). Our case emphasizes that EHE should be considered a differential diagnose of ^18^F-FDG-avid splenic malignancies.

**Patient concerns:**

A 39-year-old man presented with abdominal distension and chest distress for 20 days and lumbago for 2 days. Transthoracic echocardiography suggested a large amount of pericardial effusion. Contrast-enhanced CT imaging showed splenomegaly with multiple low-density nodules with ring enhancement. A large irregular mass was also found in the right superior mediastinum with heterogeneous density and enhancement. ^18^F-FDG PET/CT imaging revealed splenomegaly, filled with intense hypermetabolic nodules and masses. And multiple regions of increased ^18^F-FDG uptake were observed in the mediastinum, left pleura, and bones.

**Diagnosis:**

EHE of the spleen.

**Interventions:**

Half a month after the diagnosis was confirmed, the patient then underwent chemotherapy, Docetaxel combined with carboplatin, and Endu were administrated every 3 weeks.

**Outcomes:**

During the 6-month follow-up period, the patient has finished 4 cycles of chemotherapy combined with 2 months of targeted drug. Efficacy assessment is partial remission through CT imaging, and clinical symptoms of patient improved significantly.

**Lessons:**

Primary splenic EHE is extremely rare, especially with diffuse systemic metastasis. Our report suggested that EHE should be considered a differential diagnosis of ^18^F-FDG-avid splenic malignancies. Furthermore, ^18^F-FDG PET/CT plays critical role in staging and accessing disease extent of EHE.

## 1 Introduction

Epithelioid hemangioendothelioma (EHE) is a rare heterogeneous, often low-to intermediate-grade malignant vascular tumor with metastatic potential and aggressiveness.^[1]^ The incidence of EHE is about 1% per million^[2]^ and usually occurs in middle-aged. The common primary sites are liver, lung, bone, and soft tissues,^[3–5]^ while it exceptionally originates from the spleen. ^18^F-fluorodeoxyglucose positron emission tomography/computed tomography (FDG PET/CT) is advantageous in evaluating tumor activity and aggressiveness based on tumor glucose uptake. Tumors with high glucose uptake are more malignant, aggressive, and worse prognosis.^[6,7]^ Herein, we report a primary splenic EHE with diffused metastasis who underwent ^18^F-FDG PET/CT to identify the characteristics of splenic EHE in the PET/CT imaging, and to highlight the clinical importance of ^18^F-FDG PET/CT in detecting potential metastasis, accessing disease extent and staging.

## 2 Case presentation

**Figure 1:**
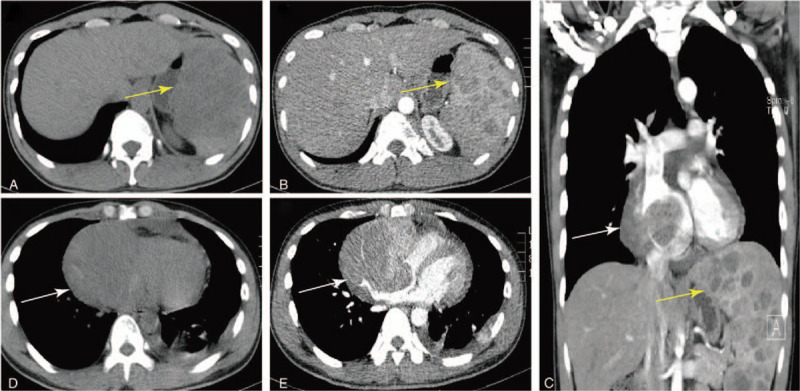
CT images (A, yellow arrow) showed splenomegaly with multiple low-density nodules with ring enhancement in contrast-enhanced CT imaging (B, C, yellow arrow). A large irregular mass was also found in the right superior mediastinum with heterogeneous density (D, white arrow) and enhancement (C, E, white arrow). CT = computed tomography.

**Figure 2:**
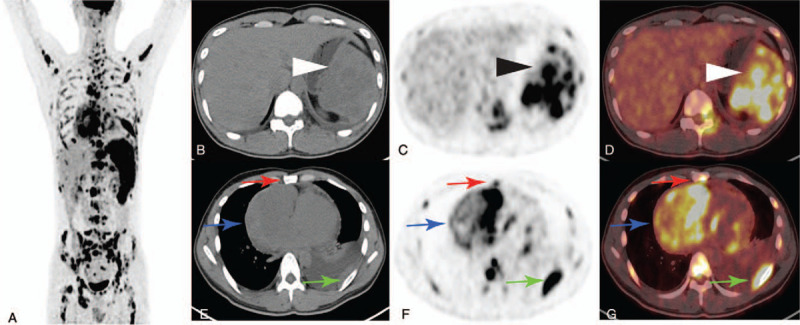
The maximum intensity projection of ^18^F-FDG PET imaging (A) revealed diffused intense hypermetabolic masses in the whole body. Transaxial PET/CT images manifested splenomegaly, filled with intense hypermetabolic nodules and masses (B–D, arrowhead). Multiple regions of increased ^18^F-FDG uptake were observed in the mediastinum (E–G, blue arrows), left pleura (green arrows), and bones (red arrows). FDG = fluorodeoxyglucose, PET/CT = positron emission tomography/computed tomography.

A 30-year-old male was admitted to our hospital with a complaint of abdominal distension, chest distress for 20 days and lumbago for 2 days. He was not an alcoholic. Physical examination revealed splenomegaly. The levels of carbohydrate antigen 125 (CA 125) and ferritin were 88.86 (normal range < 35.00 KU/L), 466.86 (normal range, 0–322.00 ng/mL), respectively. N-terminal brain linatide precursor and lactic dehydrogenase were 634.00 (normal range, 0–125.00 pg/mL), 303 (normal range, 120–250.00 pg/mL). Coagulation function test showed a normal prothrombin time and partial thromboplastin time. Liver function tests were within the normal ranges. Viral markers for hepatitis A, B, and C were negative. Transthoracic echocardiography suggested a large amount of pericardial effusion, CA 125 in pericardial effusion were elevated, which was 86.47 (normal range < 35.00 KU/L). CT images (Fig. 1A, yellow arrow) showed splenomegaly with multiple low-density nodules with ring enhancement in contrast-enhanced CT imaging (Fig. 1 B and C, yellow arrow). A large irregular mass (102 × 89 mm) was also found in the right superior mediastinum with heterogeneous density (Fig. 1D, white arrow) and enhancement (Fig. 1 C and E, white arrow). The maximum intensity projection of ^18^F-FDG PET imaging (Fig. 2A) revealed diffused intense hypermetabolic masses in the whole body (SUVmax, 20.7). Transaxial PET/CT images manifested splenomegaly, filled with intense hypermetabolic nodules and masses (SUVmax, 16.9) (Fig. 2 B–D, arrowhead). Multiple regions of increased ^18^F-FDG uptake were observed in the mediastinum (Fig. 2 E–G, blue arrows), left pleura (green arrows), and bones (red arrows).

**Figure 3:**
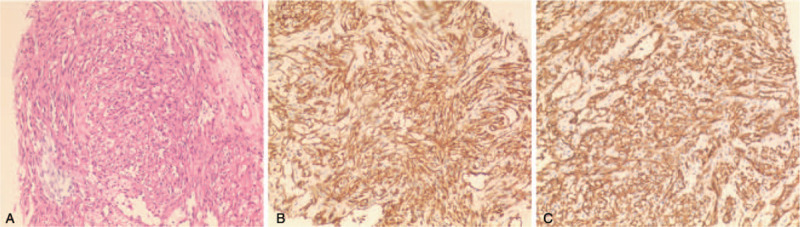
The hematoxylin-eosin staining (A, original × 100) showed tumor cells are short spindle-shaped, and vacuoles can be seen in the cytoplasm of some tumor cells. Immunohistochemical stains were strongly positive for CD31 (B, original × 100) and CD34 (C, original × 100), verifying the vascular-endothelial origin of the tumor.

Spleen biopsy was performed, and the subsequent pathological findings demonstrated the diagnosis of EHE. The hematoxylin-eosin staining (Fig. 3A, original × 100) showed tumor cells are short spindle-shaped, and vacuoles can be seen in the cytoplasm of some tumor cells. Immunohistochemical stains were strongly positive for CD31(Fig. 3B, original × 100) and CD34(Fig. 3C, original × 100), verifying the vascular-endothelial origin of the tumor. Half a month after the diagnosis, the patient started to underwent chemotherapy, Docetaxel combined with carboplatin and Endu were administrated every 3 weeks, combined with targeted drug, named Apatinib.

About 6 months later, the patient has completed 4 cycles of chemotherapy combined with 2 months of targeted drugs Apatinib. CT findings indicated that these tumors were reducing distinctly compared to before, and it evaluated the treatment efficacy as partial remission. Meanwhile, clinical symptoms have improved significantly. This case report was approved by Medical Ethical Committee of The Second Xiangya Hospital, and the informed consent of the patient was also obtained.

## 3 Discussion

EHE is a rare vascular tumor that can occur in various sites. The most common sites of EHE are liver and lung, occurrence in other parts of the body has also been reported, such as bone and soft tissue.^[8]^ Originating from the spleen is extremely rare. EHE can occur at any age, but it has a propensity to occur in the second and third decades of life,^[9]^ and it affects female than male.^[2]^ The cause of EHE is unknown, which may be associated with various factors such as exposure to asbestos, excess alcohol, and so on.^[10]^ The clinical manifestations of this disease range from abdominal pain, back pain, fever.^[11]^ Laboratory tests lack specificity, changes of blood routine, coagulation function, and tumor markers are not obvious.^[2]^

There are no specific radiographic imaging features of EHE. The tumor often appears as mixed or hypoechoic lesions on the ultrasound image, low-density lesion on the CT image, and hypointense on T1-weighted images with central enhancement and heterogeneously hyperintense on noncontrast T2-weighted images, accompanied by no or mild enhancement.^[12]^ Enhanced CT or magnetic resonance imaging could well show the pattern blood supply of the lesion. ^18^F-FDG PET/CT has a significant advantage in its ability to evaluate tumor aggressiveness, disease extent, and potential metastases due to increased glucose consumption of tumors.^[13]^ EHE needs to be differentiated from some other malignant diseases, including splenic lymphoma, metastatic carcinoma, and angiosarcoma. Splenic lymphomas are mostly secondary splenic lymphomas, a few are primary splenic lymphomas. The lesions on CT imaging show low density with mild enhancement. EHE appears as hypointense on T1-weighted images and heterogeneously hyperintense on T2-weighted images. PET/CT images showed splenomegaly with increased diffuse uptake of FDG, and enlarged lymph nodes outside of the spleen are helpful for the diagnose of splenic lymphoma.^[14]^ Metastatic carcinoma are often associated with metastases from the liver and other organs. Depending on the type of primary tumor, they could appear as clear, low-density cystic, or solid masses.^[15]^^18^F-FDG PET/CT metabolic imaging reveals radioactive concentrated lesions in multiple parts of the body. Epithelioid angiosarcoma has a high degree of malignancy. The CT images are mostly heterogeneous masses with unclear boundaries, which may be accompanied by bleeding and necrosis.^[15]^^18^F-FDG PET/CT imaging of epithelioid angiosarcoma often shows uneven FDG uptake, involving most of the spleen.^[16]^ FDG metabolism of epithelioid angiosarcoma is abnormally high, it is difficult to distinguish it from splenic EHE. The diagnosis depends on pathological and immunohistochemical examinations.

The diagnosis of EHE depends on pathology but usually hard to distinguish with sarcoma or hemangioma.^[7]^ Although EHE is classified as angiosarcomas through the recommendation of WHO, its biological behavior is unpredictable and has metastatic potential and aggressiveness. Microscopically, tumor cells form histiocyte-like morphology or epithelial cell-like arranged in small nests and cords.^[17]^ There may be vascular differentiation and intracytoplasmic vacuoles in the cytoplasm. Immunohistochemistry is necessary and helpful for the diagnosis of EHE, can be used to confirm the origin by identifying vascular endothelial cell markers, which include CD31, CD34, VIII factor, and Friend leukemia integration 1 transcription factor.^[9]^ In addition, several researches have shown 2 novel disease-defining gene fusions, named WWTR1(TAZ)-CAMTA1 and YAP1-TFE3, that were distinct subset of epithelioid hemangioendothelioma.^[18]^ The differential diagnosis for EHE includes vascular malignancies such as epithelioid angiosarcoma, and other epithelioid tumors.^[2]^

Given its rarity, clinical data to guide treatment options are limited. There is no generally accepted consensus for the treatment of HEH because of its heterogeneous status. Available treatment usually depends on the location and metastasis of disease. Surgery is the best approach for patients with local and small EHE. However, for patients of extensive, multiple lesions with distant metastasis, various promising treatment methods including chemoembolization, radiotherapy, chemotherapy, and targeted therapy have been reported.^[19–21]^ Because the clinical behavior of EHE is variable between indolent and aggressively malignant,^[9]^ it is difficult to predict the outcome; some researches have shown that tumor size > 3.0 cm and ki-67 index is related to survival.^[11,22]^^18^F-FDG PET/CT is recommended to evaluate tumor activity and to detect distant metastasis or other silent lesions, because accurate staging is crucially important for treatment and prognosis. In the present case, ^18^F-FDG PET/CT revealed the tumor was advanced stage with diffuse metastasis, which was not suitable for surgical resection. Eventually, he experienced chemotherapy and was in good condition without progress during 6 months of follow-up.

Primary splenic EHE is extremely rare, especially with diffused metastasis. Our report suggests that EHE should be considered a differential diagnosis of ^18^F-FDG-avid splenic malignancies. Furthermore, ^18^F-FDG PET/CT plays critical role in detecting potential metastasis and accessing disease extent in EHE patients.

## Author contributions

**Resources:** Jingwen Hao, Chuning Dong.

**Writing – original draft:** Xian Li.

**Writing – review & editing:** Xiaowei Ma, Yunhua Wang.

## References

[1] Weiss SW Enzinger FM. Epithelioid hemangioendothelioma: a vascular tumor often mistaken for a carcinoma. Cancer 1982;50:970–81. 7093931 10.1002/1097-0142(19820901)50:5<970::aid-cncr2820500527>3.0.co;2-z

[2] Sardaro A Bardoscia L Petruzzelli MF. Epithelioid hemangioendothelioma: an overview and update on a rare vascular tumor. Oncol Rev 2014;8:259. 25992243 10.4081/oncol.2014.259 PMC4419652

[3] Dong A Dong H Wang Y. MRI and FDG PET/CT findings of hepatic epithelioid hemangioendothelioma. Clin Nucl Med 2013;38:e66–73. 22996250 10.1097/RLU.0b013e318266ceca

[4] Rao M Chen Y Huang Z. FDG PET/CT findings of multifocal epithelioid hemangioendotheliomas of the bones. Clin Nucl Med 2015;40:821–2. 26018717 10.1097/RLU.0000000000000810

[5] Epelboym Y Engelkemier DR Thomas-Chausse F. Imaging findings in epithelioid hemangioendothelioma. Clin Imaging 2019;58:59–65. 31238187 10.1016/j.clinimag.2019.06.002

[6] Hod N Anconina R Levin D. FDG PET/CT of mediastinal epithelioid hemangioendothelioma. Clin Nucl Med 2019;44:e540–3. 31283613 10.1097/RLU.0000000000002726

[7] Watanabe S Yano F Kita T. 18F-FDG-PET/CT as an indicator for resection of pulmonary epithelioid hemangioendothelioma. Ann Nucl Med 2008;22:521–4. 18670859 10.1007/s12149-007-0159-z

[8] Weiss SW Ishak KG Dail DH. Epithelioid hemangioendothelioma and related lesions. Semin Diagn Pathol 1986;3:259–87. 3303234

[9] Rosenberg A Agulnik M. Epithelioid hemangioendothelioma: update on diagnosis and treatment. Curr Treat Options Oncol 2018;19:19. 29546487 10.1007/s11864-018-0536-y

[10] Fujii T Zen Y Sato Y. Podoplanin is a useful diagnostic marker for epithelioid hemangioendothelioma of the liver. Mod Pathol 2008;21:125–30. 18084256 10.1038/modpathol.3800986

[11] Shiba S Imaoka H Shioji K. Clinical characteristics of Japanese patients with epithelioid hemangioendothelioma: a multicenter retrospective study. BMC Cancer 2018;18:993. 30340559 10.1186/s12885-018-4934-0 PMC6194639

[12] Fan YH Tang HN Zhou JP. Fast-growing epithelioid hemangioendothelioma of the liver: a case report. Medicine (Baltimore) 2020;99:e22077. 32899078 10.1097/MD.0000000000022077 PMC7478780

[13] Tan Y Yang X Dong C. Diffuse hepatic epithelioid hemangioendothelioma with multiple splenic metastasis and delayed multifocal bone metastasis after liver transplantation on FDG PET/CT images: a case report. Medicine (Baltimore) 2018;97:e10728. 29851777 10.1097/MD.0000000000010728 PMC6392553

[14] Warshauer DM Hall HL. Solitary splenic lesions. Semin Ultrasound CT MR 2006;27:370–88. 17048453 10.1053/j.sult.2006.06.003

[15] Vancauwenberghe T Snoeckx A Vanbeckevoort D. Imaging of the spleen: what the clinician needs to know. Singapore Med J 2015;56:133–44. 25820845 10.11622/smedj.2015040 PMC4371192

[16] Zhao Q Dong A Wang Y. FDG PET/CT in primary splenic angiosarcoma with diffuse involvement of the spleen. Clin Nucl Med 2017;42:815–7. 28806241 10.1097/RLU.0000000000001805

[17] Anderson T Zhang L Hameed M. Thoracic epithelioid malignant vascular tumors: a clinicopathologic study of 52 cases with emphasis on pathologic grading and molecular studies of WWTR1-CAMTA1 fusions. Am J Surg Pathol 2015;39:132–9. 25353289 10.1097/PAS.0000000000000346 PMC4268225

[18] Antonescu CR Le Loarer F Mosquera JM. Novel YAP1-TFE3 fusion defines a distinct subset of epithelioid hemangioendothelioma. Genes Chromosomes Cancer 2013;52:775–84. 23737213 10.1002/gcc.22073 PMC4089994

[19] Zheng Z Wang H Jiang H. Apatinib for the treatment of pulmonary epithelioid hemangioendothelioma: a case report and literature review. Medicine (Baltimore) 2017;96:e8507. 29137048 10.1097/MD.0000000000008507 PMC5690741

[20] Lytle M Bali SD Galili Y. Epithelioid hemangioendothelioma: a rare case of an aggressive vascular malignancy. Am J Case Rep 2019;20:864–7. 31209195 10.12659/AJCR.915874 PMC6597141

[21] Semenisty V Naroditsky I Keidar Z. Pazopanib for metastatic pulmonary epithelioid hemangioendothelioma—a suitable treatment option: case report and review of anti-angiogenic treatment options. BMC Cancer 2015;15:402. 25967676 10.1186/s12885-015-1395-6 PMC4437555

[22] Deyrup AT Tighiouart M Montag AG. Epithelioid hemangioendothelioma of soft tissue: a proposal for risk stratification based on 49 cases. Am J Surg Pathol 2008;32:924–7. 18551749 10.1097/pas.0b013e31815bf8e6

